# Anterior Urethral Valve: Uncommon Association with Renal Duplicity

**DOI:** 10.21699/jns.v6i2.544

**Published:** 2017-04-15

**Authors:** Amina Ben Salem, Ines Mazhoud, Rachida Laamiri, Randa Salem, Hayet Laajili, Lassaad Sahnoun, Chiraz Hafsa

**Affiliations:** University of Monastir, Tunisia

**Keywords:** Anterior urethral valve, Prenatal diagnosis, Fetus, Duplex kidney

## Abstract

Anterior urethral valves (AUVs) is an unusual cause of congenital obstruction of the male urethra, being 15–30 times less common than posterior urethral valves. We present a case of AUV diagnosed at 24th gestational week. Ultrasonography and fetal MRI revealed hydronephrotic kidneys with ureteral duplicity, a distended bladder and perineal cystic mass which confirmed dilated anterior urethra in a male fetus. Diagnosis was confirmed postnatally by voiding cystourethrogram and surgery.

## Case Report

**Figure 1:**
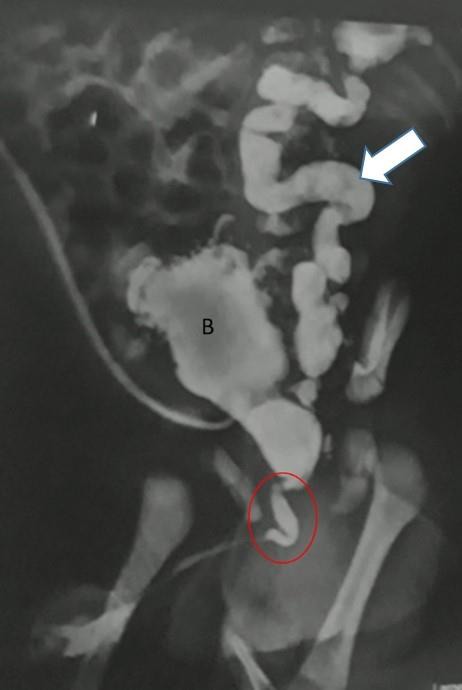
MCUG showing a distended diverticular bladder, a dilated anterior urethra consistent with anterior urethral valve (circle) and left grade V vesicoureteral reflux (arrow).

A 36-year-old woman (gravida 2, para 2) was referred to our imaging center at the 28th week of her pregnancy because of fetal hydronephrosis. Ultrasonography revealed hydronephrotic kidneys with bilateral ureteral duplicity, a distended bladder and perineal cystic mass in a male fetus. Amniotic fluid index was normal. Fetal uro-MRI showed dilated anterior urethra and bilateral hydronephrosis with a preserved renal parenchyma. Male infant was born at 38-week gestation via vaginal delivery. The newborn remained in the neonatal intensive care unit. Clinical manifestations included palpable distended bladder, and severe renal insufficiency and increased creatinine levels. Postnatal renal/bladder ultrasound demonstrated bilateral hydroureteronephrosis and showed renal duplicity. Urinary catheter passed. Voiding cystourethrogram, revealed a distended diverticular bladder, a dilated anterior urethra consistent with anterior urethral valve and left grade V vesicoureteral reflux (Fig.1). The decision was made to proceed with surgery because of renal function deterioration. Cystourethroscopy was performed. A circumferential diaphragm-type membrane was encountered in the bulbar urethra, which represented an anterior urethral valve. The AUV was resected with electrocautery. A spontaneous voiding and normal creatinine level was observed in post-operative period. Renal function evaluated by creatinine level was normal six month after operation.

Renal MAG3 scintigraphy showed a bilateral nonfunctional superior system and inferiors systems with normal renal function (left Kidney 45% and Right Kidney 55%). The child is under our strict follow-up. 

## Discussion

Congenital anterior urethral obstruction is less frequent than posterior urethral valve. However, the obstructive sequelae of AUV may be equally devastating. The association of AUV and renal duplex system is extremely rare [1]. AUV is situated mostly at the union of the glandular and penile urethra. They are most commonly found in the bulbar urethra (40%) whereas 30% present at the penoscrotal junction and 30% at the pendulous urethra [2]. Patients with AUV that are diagnosed prenatally, usually present with bilateral hydronephrosis and in severe cases with mega-ureters and/or megacystis. In our case ultrasound revealed bilateral hydronephrosis, distended bladder and cystic dilatation of anterior urethra. Prenatal MRI appears as a useful complementary diagnostic in complex case and helps in evaluation of renal dysplasia [3].

Voiding cystourethrogram (VCUG) remains the gold standard imaging modality for the diagnosis of urethral anomalies. Typical findings on VCUG include a thickened trabeculated bladder and dilated anterior urethra. Transurethral valve ablation, either with laser, electro cautery, or cold-cut, is a very effective way owing to miniaturized instruments made available [2]. Anterior urethral valve is a rare cause of bladder outlet obstruction. It can present prenatally with features of bladder outlet obstruction. Postnatal management is similar to patients with PUV. However accurate description of valve can be given only after a careful cystourethroscopy.

## Footnotes

**Source of Support:** None

**Conflict of Interest:** None
